# A Novel Teaching-Learning-Based Optimization with Error Correction and Cauchy Distribution for Path Planning of Unmanned Air Vehicle

**DOI:** 10.1155/2018/5671709

**Published:** 2018-08-01

**Authors:** Zhibo Zhai, Guoping Jia, Kai Wang

**Affiliations:** College of Mechanical and Equipment Engineering, Hebei University of Engineering, Handan, Hebei 056038, China

## Abstract

Teaching-learning-based optimization (TLBO) algorithm is a novel heuristic method which simulates the teaching-learning phenomenon of a classroom. However, in the later period of evolution of the TLBO algorithm, the lower exploitation ability and the smaller scope of solutions led to the poor results. To address this issue, this paper proposes a novel version of TLBO that is augmented with error correction strategy and Cauchy distribution (ECTLBO) in which Cauchy distribution is utilized to expand the searching space and error correction to avoid detours to achieve more accurate solutions. The experimental results verify that the ECTLBO algorithm has overall better performance than various versions of TLBO and is very competitive with respect to other nine original intelligence optimization algorithms. Finally, the ECTLBO algorithm is also applied to path planning of unmanned aerial vehicle (UAV), and the promising results show the applicability of the ECTLBO algorithm for problem-solving.

## 1. Introduction

Global optimization is a universal issue to the entire scientific community. It has been applied widely in many different fields such as chemical engineering [1], molecular biology [2], the training of neural networks [3], job shop scheduling [4], and network design [5]. However, in most cases, global optimization problems are nonlinear and nondifferentiable and, hence, gradient-based methods cannot be used. In recent years, a lot of effective optimization algorithms have been developed and used to successfully solve global optimization problems that are nonlinear and nondifferentiable. Typical algorithms include particle swarm optimization (PSO) [6] proposed by Kennedy and Eberhart in 1995 and inspired by swarm behavior of fish schooling and bird flocking, differential evolution (DE) [7] which mimics Darwinian evolution, group search optimizer (GSO) [8] which is inspired by animal searching behaviors, artificial bee colony (ABC) [9] which simulates the foraging behavior of honey bees, water cycle algorithm (WCA) [10] which is based on the observation of water and cycle processes and how rivers and streams flow to the sea in the real world, cuckoo search (CS) [11] which mimics the brooding behavior of some cuckoo species, backtracking search algorithm (BSA) [12] which is developed from the differential evolution algorithm, differential search algorithm (DSA) [13] which is inspired by the migration of superorganisms utilizing the concept of stable motion, and interior search algorithm (ISA) [14] which is inspired by interior design and decoration.

Recently, Rao et al. [15] proposed the teaching-learning-based optimization (TLBO) algorithm inspired by the teaching-learning process in a classroom. The algorithm simulates two fundamental phases of learning consisting of the “Teacher Phase” and the “Learner Phase.” One of the remarkable advantages of the TLBO algorithm is its simple computation. The other important advantage of the TLBO algorithm is that it does not require specific controlling parameters (the crossover and mutation probability, etc.) except for the common controlling parameters (the size of population and the problem dimensional), which makes the TLBO algorithm easy to implement and more quickly convergence speed. Hence, it has been extended to engineering optimization [15], physics-biotechnology optimization [16], multiobjective optimization [17], heat exchanger design [18], dynamic economic emission dispatch [19], and so on.

Although the TLBO algorithm has some advantages, it has some undesirable dynamical properties that degrade its searching ability. One of the most important issues is that there exists the lower exploitation ability and the smaller scope of solutions in the later stages of evolution. Another issue is regarding the ability of the TLBO algorithm to balance exploration and exploitation [20]. Exploration is the ability that the TLBO algorithm develops global solution space, and the exploitation is the ability that the TLBO algorithm searches the approximately optimization solution in local solution space. Overemphasize of exploration process prevents the population converging, while too much emphasis on the exploitation process tends to cause the premature convergence of the population. In practice, the exploration and exploitation processes contradict each other and, in order to achieve good solutions, the two processes should be properly trade-off. To improve the performance of the TLBO, modified or improved algorithms are proposed in recent years, such as an elitist teaching-learning-based optimization (ETLBO) algorithm [21], teaching-learning-based optimization with neighborhood search (NSTLBO) [22], and teaching-learning-based optimization with dynamic group strategy (DGSTLBO) [20].

Although the modified TLBO algorithms have better performance than the TLBO for some classical problems, some important issues are not considered such as there are still the lower exploitation ability and the smaller scope of solutions in the later stages of evolution. To address these issues, this paper proposes a novel version of TLBO that is augmented with error correction and Cauchy distribution (ECTLBO) in which the Cauchy distribution is utilized to expand the searching space and error correction to avoid detours to achieve more accurate solutions.

The rest of this paper is organized as follows.  Section 2 first briefly introduces the TLBO algorithm and the details of its implementation.  Section 3 presents TLBO with error correction and Cauchy distribution (ECTLBO).  Section 4 analyzes the results of ECTLBO and several related optimization algorithms via a comparative study.  Section 5 applies ECTLBO algorithm to path planning of unmanned aerial vehicle (UAV). Finally, the work is summarized in Section 6.

## 2. Teaching-Learning-Based Optimization

The teaching-learning-based optimization algorithm is a nature-inspired algorithm analogous to the teaching-learning process in a class between a teacher and learners. The process of implementing TLBO consists of two phases, “Teacher Phase” and “Learner Phase.” The “Teacher Phase” stands for learning from the teacher while the “Learner Phase” denotes learning through the interaction between learners.

### 2.1. Teacher Phase

During the Teacher Phase, the updating formula of the learning for a learner *X*_*i*_ (*i* = 1, 2, *N*, where *N* is the number of learners), *X*_*i*_ is a vector of a learner which includes *x*_*ij*_ consisting of various subjects such as literature, mathematics, and English (*j* = 1, 2, *D*, *X*_*i*_ (*x*_*i*1_, *x*_*i*2_,, *x*_*iD*_, where *D* is the number of subjects which a learner *X*_*i*_ studied)), is

(1) $$\begin{array}{c} X_{i,\text{new}}=X_{i}+r\left(X_{\text{teacher}}-T_{F}X_{\text{mean}}\right), \end{array}$$

where *X*_*i*,new_ is a newly generated individual according to *X*_*i*_, *X*_*t*_ is the best individual of current population, *X*_mean_ is the current mean value of all individuals, *r* is a vector whose elements are distributed randomly in [0, 1], and *T*_F_ is a teaching factor deciding the value of the *X*_mean_ to be changed. The value of *T*_F_ is either 1 or 2, indicating the learner learns something or nothing, respectively, from the teacher. The value of *T*_F_ is decided randomly with equal probability:

(2) $$\begin{array}{c} T_{F}=\text{round}\left[1+\mathrm{rand}\left(0,1\right)\right]. \end{array}$$

### 2.2. Learner Phase

During the Learner Phase, each learner interacts with other learners to improve his or her knowledge. A learner *X*_*i*_ learns something new if the other learner *X*_*j*_ has more knowledge than him or her. *f*(*X*_*i*_) is the summary of all the scores of subjects for the *i*th learner, and the updating formula of the learning for a learner *X*_*i*_ is

(3) $$\begin{array}{c} X_{i,\text{new}}=\left\{\begin{array}{cc} X_{i}+r\left(X_{i}-X_{j}\right), & \text{if }f\left(X_{i}\right)<f\left(X_{j}\right),\\ X_{i}+r\left(X_{j}-X_{i}\right), & \text{if }f\left(X_{j}\right)<f\left(X_{i}\right). \end{array}\right. \end{array}$$

## 3. A Novel Teaching-Learning-Based Optimization

In this study, a novel version of TLBO that is augmented with an error correction strategy and Cauchy distribution (ECTLBO) is proposed.

### 3.1. Error Correction Strategy

Some learners who have a bad result because of the bad study method should be guided correctly. Because the study method of some learners toward the teacher is wrong, this time if this is not corrected in time, there will be a detour phenomenon. The study method has problems even wrong, and this leads to opposite. Although each learner spends a lot of efforts, the effect is not too obvious. So, it must have correction function to avoid detours to achieve faster convergence speed and the precision of the optimization as long as the learner who has back phenomenon is corrected in a timely manner. The updating equation of the Teacher Phase is

(4) $$\begin{array}{c} X_{i,\text{new}}=X_{i}+\text{Cauchy}\left(0,1\right)\;*\;\left(X_{\text{teacher}}-T_{F}X_{\text{mean}}\right),\\ X_{i,\text{new}\_c}=X_{i}-\text{Cauchy}\left(0,1\right)\;*\;\left(X_{\text{teacher}}-T_{F}X_{\text{mean}}\right). \end{array}$$

### 3.2. Cauchy Distribution

Cauchy distribution is a common distribution in probability theory and mathematical statistics, and the probability density function in dimension is as follows:

(5) $$\begin{array}{c} f\left(x_{1},x_{2},\ldots ,x_{D}\right)=\frac{t}{\pi \left(t+X^{2}\right)},\;X=\left(x_{1},x_{2},\ldots ,x_{D}\right),t\in R. \end{array}$$

**Figure 1:**
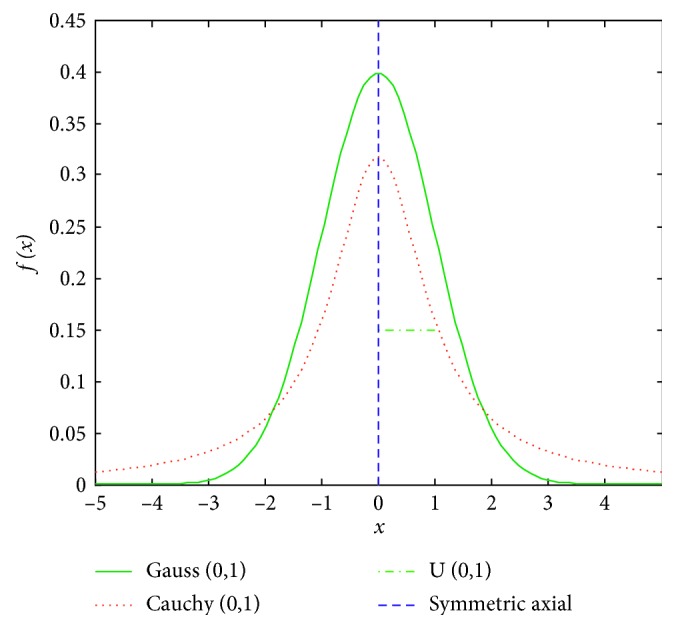
Probability density curves of standard Gauss, Cauchy, and uniform distributions.

It is the standard Cauchy distribution when the parameter *t* equals 1.  Figure 1 is the probability density curves of standard Gauss distribution, standard Cauchy distribution, and standard uniform distribution, respectively. As can be seen from Figure 1, the peak of Cauchy distribution at the origin is the smallest of three different distributions, while the velocity of the long flat shape near to zero is the slowest. So, if the mutation strategy of Cauchy distribution is used in the Teacher Phase and Learner Phase, its disturbance ability or self-adjustment ability is the strongest of three different distributions, and the basic TLBO algorithm is more likely to jump out of the local optimum and improve the search speed. The updating equation of the Learner Phase is

(6) $$\begin{array}{c} X_{i,\text{new}}=\left\{\begin{array}{cc} X_{i}+\text{Cauchy}\left(0,1\right)\left(X_{i}-X_{j}\right), & \text{if }f\left(X_{i}\right)<f\left(X_{j}\right),\\ X_{i}+\text{Cauchy}\left(0,1\right)\left(X_{j}-X_{i}\right), & \text{if }f\left(X_{j}\right)<f\left(X_{i}\right). \end{array}\right. \end{array}$$

### 3.3. Flowchart of Distribution of ECTLBO Algorithm

**Figure 2:**
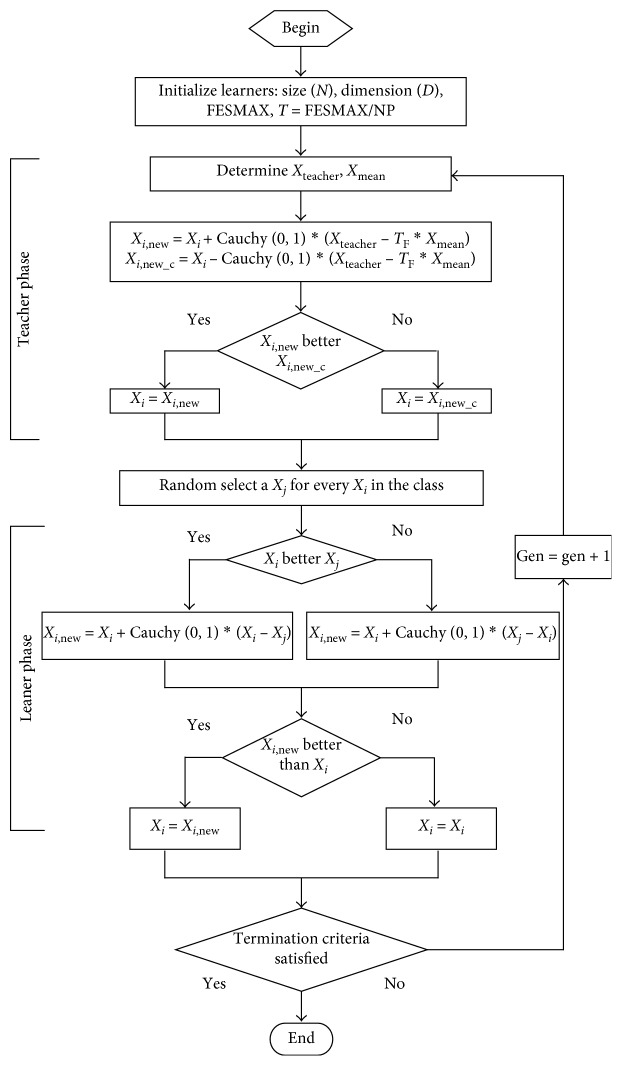
Flowchart of ECTLBO algorithm.

As explained above, the flowchart of an error correction strategy and Cauchy distribution (ECTLBO) is shown in Figure 2.

## 4. Experimental Studies

### 4.1. Test Benchmark Functions

To evaluate the performance of the ECTLBO algorithm, 6 contest benchmark functions [23] are used in a set of experimental studies. The definition of these functions is given in Table 1.

### 4.2. Experimental Platform and Termination Criterion

All experiments are conducted on the same computer with a Celoron 2.26 GHz CPU, 2 GB memory, and windows XP operating system with MATLAB 7.9. For the purpose of decreasing statistical errors, all experiments are repeated 25 times for all 6 test functions of 30 dimensions. Also, 300,000 function evaluations (FEs) [24] are used as the stopping criterion.

### 4.3. Performance Metric

The mean value (*F*_mean_) of the function error value *f*(*X*)−*f*(*X*^*∗*^) is recorded to evaluate the performance of each algorithm, where *f*(*X*) and *f*(*X*^*∗*^) denote the best fitness value for solution and the real global optimization value of the test problem, respectively. The standard deviation (SD) indicates robust of various optimization algorithms on *F*_1_–*F*_6_ test functions on 30 dimensions. To verify whether the overall optimization performance of various optimization algorithms is significantly different, statistical analysis is used to compare the results obtained by the algorithms for the same kind of problems. Therefore, the statistical tool Wilcoxon's rank sum test [25] at a 0.05 significance level is adopted. The Wilcoxon's rank sum test assesses whether the mean value (*F*_mean_) of two solutions from any two algorithms is statistically different from each other.

### 4.4. Comparison of ECTLBO with Relevant TLBO Algorithms

**Figure 3:**
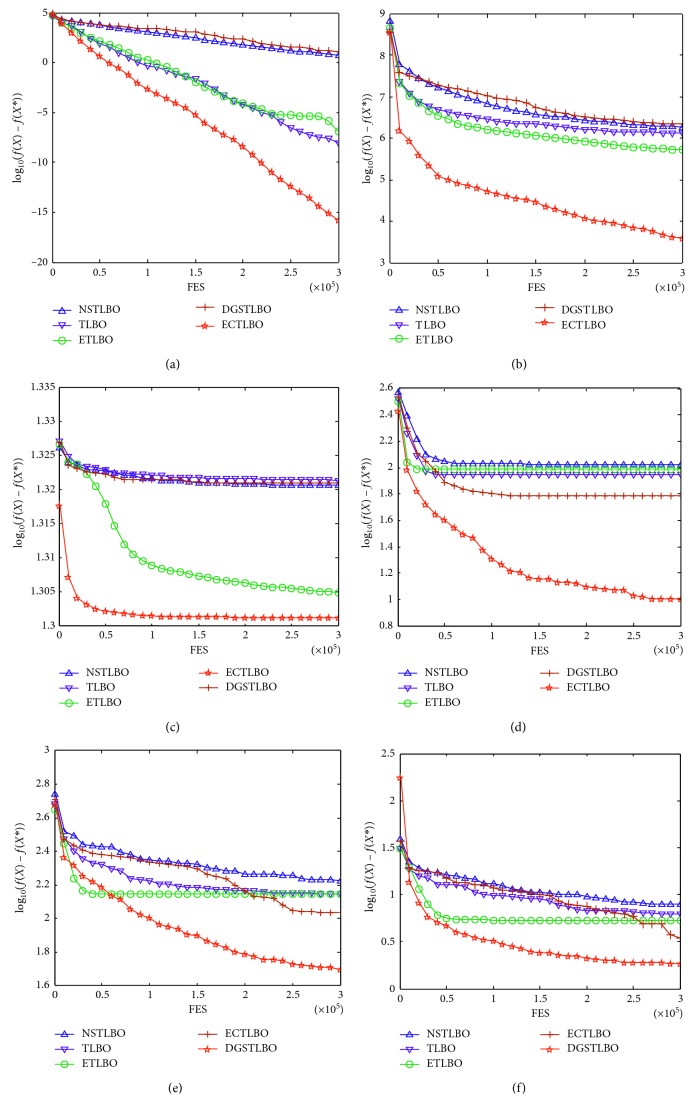
Evolution of mean function error values derived from five algorithms: (a) *F*_1_, (b) *F*_2_, (c) *F*_3_, (d) *F*_4_, (e) *F*_5_, and (f) *F*_6_.

The ECTLBO algorithm is compared to four different relevant TLBO algorithms: TLBO, ETLBO, NSTLBO, and DGSTLBO. The parameters for four relevant TLBO algorithms are taken from their references listed above. Each algorithm runs independently 25 times, and the statistical results of *F*_mean_ and SD are provided in Table 2, the last three rows of which show the experimental results, and the best results are shown in bold. The evolution plots of NSTLBO, TLBO, ECTLBO, ETLBO, and DGSTLBO are illustrated in Figure 3. In addition, the semilogarithmic convergence plots are used to analyze the relationship of the mean errors of the functions.

In this section, the ECTLBO algorithm is compared with four relevant TLBO algorithms. From the statistical mean value (*F*_mean_) given in Table 2, the overall performance of the ECTLBO algorithm is significantly better than that of other algorithms. The ECTLBO algorithm outperforms NSTLBO, TLBO, ETLBO, and DGSTLBO on six, six, five, and six test functions out of six test functions, respectively. As can be seen from the statistical mean value (*F*_mean_) in Table 2, the ECTLBO algorithm is better than the other four algorithms for functions *F*_1_, *F*_2_, *F*_4_, *F*_5_, and *F*_6_. For function *F*_3_, the ECTLBO algorithm performs the same as the ETLBO algorithm in terms of the statistical mean value (*F*_mean_). Therefore, it is interesting to note that the overall performance of the ECTLBO algorithm is significantly better than the original TLBO, ETLBO, NSTLBO, and DGSTLBO algorithms.

Considering the above situations, the main reason is that the Cauchy distribution can expand the searching space and error correction to avoid detours to achieve more accurate solution, helping to identify a more promising solution. That is to say, exploration and exploitation are balanced better in the ECTLBO algorithm. Therefore, it can be concluded that the ECTLBO algorithm performs most effectively for accuracy among the five relevant TLBO algorithms.

### 4.5. Comparison of ECTLBO Algorithm with Nine Original Intelligence Optimization Algorithms

In this section, the ECTLBO algorithm is compared with nine original intelligence optimization algorithms including PSO [6], DE [7], GSO [8], ABC [9], WCA [10], CS [11], BSA [12], DSA [13], and ISA [14]. From the statistical mean value (*F*_mean_) of Table 3, it can be seen that the ECTLBO algorithm performs better than other nine original intelligence optimization algorithms based on the Wilcoxon's rank sum test results. More specifically, the ECTLBO algorithm outperforms PSO, DE, ABC, CS, GSO, WCA, DSA, BSA, and ISA algorithms on five, six, three, six, three, five, four, three, and four out of six test functions, respectively. For unimodal functions *F*_1_ and *F*_2_, the ECTLBO algorithm especially outperforms above other nine original intelligence optimization algorithms. As can be seen from the standard deviation (SD) of Table 3, the ECTLBO algorithm is better than nine original intelligence optimization algorithms on functions *F*_1_, *F*_2_, and *F*_6_. This indicates that robustness of the ECTLBO algorithm is better than that of nine original intelligence optimization algorithms on functions *F*_1_, *F*_2_, and *F*_6_.

## 5. Application of ECTLBO to UAV Path Planning

### 5.1. Path Planning of Unmanned Aerial Vehicles (UAV) Problem

**Figure 4:**
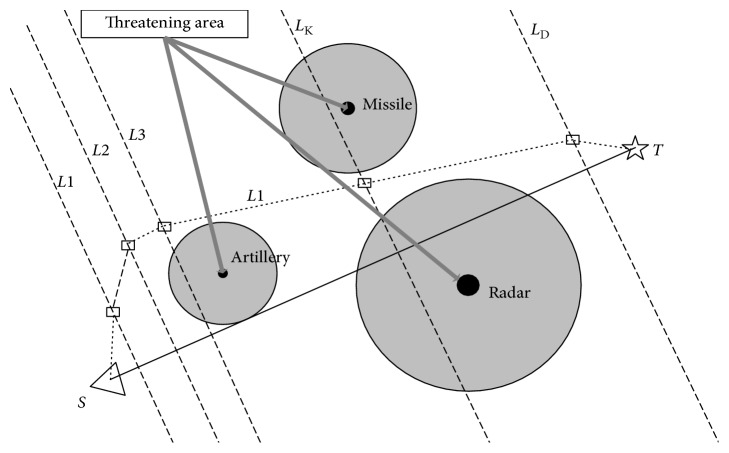
Typical UAV battle field model.

UAV is a rather complicated global optimum problem in mission planning. This problem aims to look for or figure out an optimal or suboptimal flight route from the starting point to the target under specific complex combat field environment in time [26].  Figure 4 shows that the problem of UAV is considered as *D*-dimensional function optimization problem in essence. The original coordinate system O*xy* is converted to a new coordinate system O*x*′*y*′. The axis *x*′ is divided equally into *D* parts, the *y*′ coordinates of each node on the vertical line are optimized, and a set of points composed of vertical coordinates of *D* points is obtained. By connecting these points in sequence, we get a path connecting the starting point and the destination.

There are two main goals of UAV path planning: avoiding threats and minimizing fuel costs. Therefore, before studying UAV route optimization, we must determine the performance indicators of each path. The following cost equations [27] are used to describe the minimum threat cost and minimum fuel safety performance:

(7) $$\begin{array}{c} \min J=\int_{0}^{J}\left[kw_{t}+\left(1-k\right)w_{f}\right]d_{s}, \end{array}$$

where *L* is the length of the route, *J* is a generalized cost function, *w*_t_ is a threat cost, *w*_f_ is the cost of oil consumption, and the coefficient *k* ∈ [0,1] is the trade-off factor of the threat factor and the performance of fuel consumption.

According to the path planning performance index formula, the cost weight of each edge in the network diagram is calculated. For the feasible UAV route of section *i*, the cost weight value can be expressed as

(8) $$\begin{array}{c} w_{i}=kw_{t,i}+\left(1-k\right)w_{f,i},\;\left(0\leq k\leq 1\right). \end{array}$$

**Figure 5:**
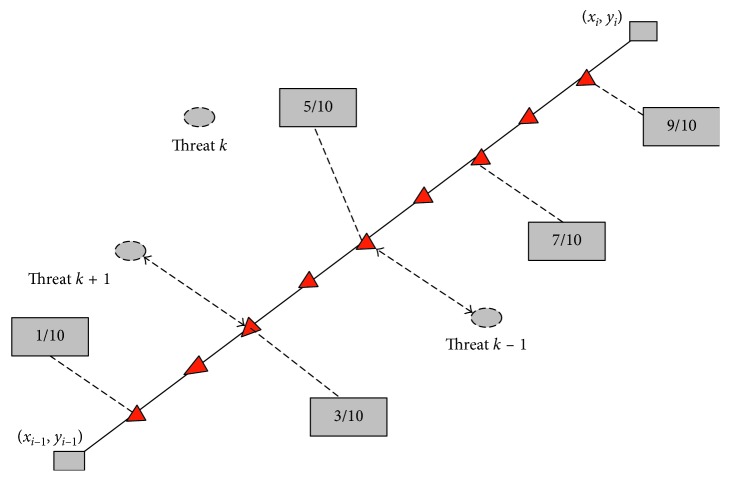
Threat cost model.

It is assumed that all radars in the enemy defense area are the same and not interconnected, and the radar threat model is simplified, and the radar signal is proportional to 1/*d*^4^ (*d* indicates the distance between the UAV to the enemy radar and the missile threat position), so the threat cost between the two nodes when the UAV is flying along the line *i* of the network map. The approximation is considered to be proportional to the integral of 1/*d*^4^ along this edge (as shown in Figure 5). In simulation studies, it is usually simplified to divide the segment into five segments within the threat:

(9) $$\begin{array}{c} w_{i,L_{ij}}=\frac{L_{ij}^{5}}{5}\overset{N_{t}}{\underset{k=1}{\sum }}t_{k}\times \left(\frac{1}{d_{0.1,k}^{4}}+\frac{1}{d_{0.3,k}^{4}}+\frac{1}{d_{0.5,k}^{4}}+\frac{1}{d_{0.7,k}^{4}}+\frac{1}{d_{0.9,k}^{4}}\right), \end{array}$$

where *L*_*ij*_ represents the length of the connection point; *d*_0.1,*k*_, *d*_0.3,*k*_, *d*_0.5,*k*_, *d*_0.7,*k*_, and  *d*_0.9_ show distances from the center *k* of the threat source at 0.1, 0.3, 0.5, 0.7, and 0.9; *t*_*k*_ represents the threat weight of *k* threats; and *N*_t_ is the number of threat positions.

### 5.2. Analysis of Simulation Results and Comparisons

**Figure 6:**
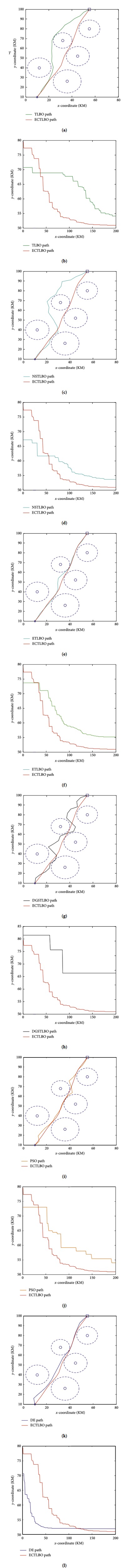
Optimal flight routes and evolutionary curves derived from seven algorithms.

Table 4 gives the threat points of the UAV and the coordinates of the starting point and the target point. By selecting the appropriate parameters, the classical algorithms such as ECTLBO and TLBO, NSTLBO, ETLBO, DGSTLBO, PSO, and DE are applied to the UAV path planning, and the simulation results are shown in Figure 6, respectively. As can be seen from Figure 6, TLBO, NSTLBO, ETLBO, and DGSTLBO all fall into local optimal. Although PSO and DE do not fall into the local optimal, the optimal route map generated by these two algorithms is obviously longer than that of the algorithm ECTLBO. At the same time, it can be seen from Figure 6 that the unmanned aerial vehicle (UAV) route obtained by the ECTLBO algorithm has successfully avoided all the threat sources and successfully reached the task end. By comparing with TLBO, NSTLBO, ETLBO, DGSTLBO, PSO, and DE algorithms, the experimental results show that the ECTLBO algorithm can get higher quality navigation trace and higher quality convergence and better avoid the threat route. And the convergence speed is faster than other classical algorithms.

## 6. Summary and Conclusions

This paper presents a new version of the TLBO algorithm (ECTLBO), in which error correction and Cauchy distribution are introduced. The performance of the ECTLBO algorithm is evaluated compared with that of other variant TLBO algorithms, nine original intelligence optimization algorithms. The experimental results verify that the ECTLBO algorithm has overall better performance than that of other variant TLBO algorithms and is very competitive among them. Besides that, we also applied it to UAV, and the simulation results show that the path planning stability and path quality by the proposed approach are much more smooth, and shorter and more optimal than those by other well-known algorithms.

## Figures and Tables

**Table 1:** Definition and optimum of benchmark functions used to evaluate optimization algorithms.

Name	Definition	Optimum
*F*_1_	Shifted Schwefel's problem 1.2 (first version for a second revision of Schwefel's function)	0
*F*_2_	Shifted, rotated, high-conditioned elliptic function	0
*F*_3_	Shifted, rotated Ackley's function with global optimum on bounds	0
*F*_4_	Shifted Rastrigin's function	0
*F*_5_	Shifted, rotated Rastrigin's function	0
*F*_6_	Expanded, extended Griewank's plus Rosenbrock's function	0

**Table 2:** Results of five algorithms for 25 independent runs on 6 test functions of 30 dimensions.

Function	Result	TLBO	DGSTLBO	ETLBO	NSTLBO	ECTLBO
*F*_1_	*F*_mean_	5.26*E* − 10−	4.52*E* − 00−	4.52*E* − 10−	6.03*E* − 00−	**9.60*E* − 15**
	SD	9.89*E* − 10	5.01*E* − 00	2.26*E* − 09	2.22*E* − 00	1.48*E* − 10

*F*_2_	*F*_mean_	1.07*E* + 06−	3.35*E* + 06−	2.83*E* + 04−	8.87*E* + 06−	8.73*E* + 05
	SD	7.16*E* + 05	1.42*E* + 06	1.41*E* + 05	4.94*E* + 05	3.30*E* + 05

*F*_3_	*F*_mean_	2.09*E* + 01−	2.10*E* + 01−	2.04*E* + 01≈	2.09*E* + 01−	**2.03*E* + 01**
	SD	4.00*E* − 02	5.42*E* − 02	4.03*E* + 00	6.34*E* − 02	5.39*E* − 02

*F*_4_	*F*_mean_	8.59*E* + 01−	6.37*E* + 01−	1.44*E* + 02−	1.38*E* + 02−	**1.23*E* + 01**
	SD	1.92*E* + 01	2.59*E* + 01	4.56*E* + 01	2.55*E* + 02	2.04*E* + 01

*F*_5_	*F*_mean_	1.23*E* + 02−	9.04*E* + 02−	2.14*E* + 02−	2.03*E* + 02−	**1.19*E* + 02**
	SD	3.30*E* + 01	5.50*E* + 01	3.61*E* + 01	2.97*E* + 01	1.53*E* + 01

*F*_6_	*F*_mean_	4.32*E* + 00−	4.50*E* + 00−	3.67*E* + 00−	7.45*E* + 00−	**3.36*E* + 00**
	SD	1.02*E* + 00	3.27*E* + 00	7.93*E* − 01	2.73*E* + 00	6.28*E* − 01

	−	6	6	5	6	
	+	0	0	0	0	
	≈	0	0	1	0	

“−,” “+,” and “≈”denote that the performance of the corresponding algorithm is significantly worse than, significantly better than, and similar to that of ECTLBO, respectively.

**Table 3:** Results of ten algorithms over 25 independent times on 6 test functions of 30 dimensions with 300,000 FES.

Function	Result	PSO	DE	ABC	CS	GSO	WCA	DSA	BSA	ISA	ECTLBO
*F*_1_	*F*_mean_	3.15*E* + 03−	4.85*E* − 14−	8.19*E* + 03−	2.48*E* − 00−	8.83*E* + 02−	4.81*E* − 01−	1.38*E* + 03−	1.08*E* + 02−	2.22*E* − 04−	9.60*E* − 15
	SD	4.22*E* + 03	1.16*E* − 13	1.82*E* + 03	1.06*E* − 00	1.71*E* + 02	4.00*E* − 01	4.88*E* + 02	4.82*E* + 01	6.16*E* − 04	1.48*E* − 10

*F*_2_	*F*_mean_	2.50*E* + 07−	9.23*E* + 05−	9.01*E* + 06−	3.36*E* + 06−	2.17*E* + 06−	1.74*E* + 06−	1.38*E* + 07−	1.88*E* + 06−	1.18*E* + 07−	8.73*E* + 05
	SD	2.53*E* + 07	4.56*E* + 05	1.95*E* + 06	7.40*E* + 05	5.19*E* + 05	6.20*E* + 05	5.67*E* + 06	7.10*E* + 05	5.36*E* + 06	3.30*E* + 05

*F*_3_	*F*_mean_	2.09*E* + 01−	2.10*E* + 01−	2.03*E* + 01≈	2.09*E* + 01−	2.03*E* + 01≈	2.04*E* + 01≈	2.09*E* + 01−	2.09*E* − 01−	2.03*E* + 01≈	2.03 *E* + 01
	SD	6.47*E* − 02	4.20*E* − 02	4.60*E* − 02	6.97*E* − 02	1.08*E* − 01	6.57*E* − 02	5.36*E* − 02	5.52*E* − 02	1.17*E* − 01	5.39*E* − 02

*F*_4_	*F*_mean_	7.51*E* + 01−	1.90*E* + 01−	0+	6.09*E* + 01−	7.76*E* − 00+	4.90*E* + 01−	0+	3.61*E* − 14+	1.17*E* + 02+	1.23*E* + 01
	SD	3.28*E* + 01	4.28*E* − 00	0	7.96*E* − 00	3.00*E* − 00	1.55*E* + 01	0	1.14*E* − 13	2.76*E* + 01	2.04*E* + 01

*F*_5_	*F*_mean_	1.25*E* + 02−	1.28*E* + 02−	3.39*E* + 02−	1.39*E* + 02−	3.21*E* + 02−	1.57*E* + 02−	9.54*E* + 02−	8.34*E* + 01+	1.53*E* + 024	1.19*E* + 02
	SD	3.31*E* + 01	6.77*E* + 01	4.87*E* + 01	1.77*E* + 01	7.18*E* + 01	4.05*E* + 01	1.18*E* + 01	1.55*E* + 01	97*E* + 01	1.53*E* + 01

*F*_6_	*F*_mean_	3.36*E* − 00≈	2.94*E* − 00−	1.13*E* − 00+	6.74*E* − 00−	2.10*E* − 00+	9.86*E* − 00−	1.85*E* − 00+	1.75*E* − 00+	4.42*E* − 00−	3.36*E* + 00
	SD	7.40*E* − 01	7.24*E* − 01	9.39*E* − 01	7.47*E* − 01	4.53*E* − 01	2.02*E* − 00	8.90*E* − 01	1.29*E* − 00	1.15*E* − 00	6.28*E* − 01

	−	5	6	3	6	3	5	4	3	4	
	+	0	0	2	0	2	0	2	3	1	
	≈	1	0	1	0	1	1	0	0	1	

“−,” “+,” and “≈”denote that the performance of the corresponding algorithm is significantly worse than, significantly better than, and similar to that of ECTLBO, respectively.

**Table 4:** The parameters of threat environment.

Start point	(10, 10)					
Target point	(55, 100)					
	Number	Threat center \threat radius \threat level	Number	Threat center \threat radius \threat level	Number	Threat center \threat radius \threat level

Threat point	1	(12, 40)\10\10	2	(32, 68)\8\1	3	(36, 26)\12\2
	4	(45, 50)\10\2	5	(55, 80)\9\3	—	—

Weight value	*k* = 0.5					

## Data Availability

The data used to support the findings of this study are available from the corresponding author upon request.
